# Development and validation of a facile rapid assessment scale for perinatal venous thromboembolism in puerperium in Chinese women

**DOI:** 10.1186/s12884-023-05901-1

**Published:** 2023-08-24

**Authors:** Xiujuan Chen, Wei Peng, Yan Zhang, Huansheng Zhou, Meng Zhang

**Affiliations:** 1 Nursing Department, the Affiliated Hospital of Qingdao University, Qingdao, China; 2 Department of Obstetrics, the Affiliated Hospital of Qingdao University, Qingdao, China

**Keywords:** Risk factors, Risk assessment scale, Perinatal venous thromboembolism, Pregnant woman, Full-term parturient women

## Abstract

**Background:**

It is still urgent and challenge to develop a simple risk assessment scale for venous thromboembolism (VTE) in puerperium in Chinese women.

**Methods:**

The study, a retrospective case-control study, was conducted in 12 hospitals in different cities in China. A total of 1152 pregnant women were selected, including 384 cases with VTE and 768 cases without VTE. A logistic regression method was conducted to determine the risk factors of VTE.

**Results:**

Age, BMI before delivery, gestational diabetes mellitus, family history (thrombosis, diabetes, cardiovascular disease), and assisted reproductive technology were independent risk factors (P<0.05). The difference between the high-risk group and the low-risk group was statistically significant(P<0.001) with a sensitivity of 0.578, specificity of 0.756, Yuden index o.334, and area under the ROC curve of 0.878.

**Conclusions:**

The age (≥ 35 years), BMI before delivery (≥ 30 kg/m^2^), gestational diabetes mellitus, family history of related diseases and assisted reproductive technology are more likely to cause VTE after full-time delivery. The simple and rapid assessment scale of VTE in women after full-term delivery has perfect discrimination (P < 0.001), which can be applied to predict the risk of VTE in Chinese full-term postpartum women.

## Introduction

Venous thromboembolism (VTE) remains a major cause of maternal morbidity and mortality in the western world, which manifests as pulmonary embolism (PE) or deep vein thrombosis (DVT)[1–5]. The perinatal period places women at risk of developing venous thromboembolism (VTE). Venous thromboembolism can occur at any stage of pregnancy (pregnancy, delivery and puerperium) but the puerperium is the time of highest risk [6]. Women with VTE during pregnancy suffer long-term sequelae like chronic edema, skin changes, recurrent thrombosis and ulceration [7]. The overall prevalence of VTE during pregnancy is approximately 2 per 1000 deliveries. During pregnancy, the risk of VTE is 4–5 fold compared to non-pregnant women while the risk is 10 times higher during the postpartum period. VTE accounts for 1.1 deaths per 100 000 deliveries or 10% of all maternal deaths [8–11]. Although the absolute VTE rates are relatively low, pregnancy-associated VTE is an important cause of maternal morbidity and mortality. Given the high maternal mortality due to VTE, early diagnosis and treatment should be prioritized [12]. However, the subjective clinical assessment of deep venous thrombosis (DVT) and pulmonary embolism (PE) is particularly unreliable in pregnancy. Only a minority of women with clinically suspected VTE are diagnosed with VTE when objective testing is employed. It is urgent to explore the risk factors for VTE and develop a facile rapid assessment scale in puerperium in Chinese women to provide an applicable and reliable assessment method.

The VTE risk assessment tools have been extensively investigated in western countries and a series of assessment scales have been provided [13–18]. For example, Dargaud et al. provided a Lyon VTE risk prediction scale in 2005 and modified this Lyon score in a multicenter clinical trial in 2010 in France [19–21]. Chauleur et al. designed a STRATHEGE rating scale in 2008 and they updated the scale in 2018 [22, 23]. In 2011, Schoenbeck et al. from the UK presented a scoring system [24]. Italian Sophie Testa et al. developed an assessment of the risk of VTE named Pregnancy Health-care Program (PHP) in 2015 [25]. In 2012, American College of Chest Physicians (ACCP) issued *VTE, thrombophilia, antithrombotic therapy, and pregnancy guidelines* [26]. In 2018, American Society of Hematology (ASH) released *guidelines for management of venous thromboembolism in pregnancy* [27]. In April 2015, the Royal College of Obstetricians and Gynaecologists (RCOG) released an updated version of the clinical guidelines for the risk of VTE during pregnancy and puerperium [3]. However, there are some lacks in the currently available tools for pregnancy and the postpartum period. For the guidelines of RCOG, the maternal and fetal radiation risks of tests used in the diagnosis of PE in pregnancy need to be clarified. According to the guidelines of ACCP and RCOG, mothers with VTE scores ≥ 2 points in puerperal period should be used low molecular weight heparin (LMWH) to prevent VTE. But it is difficult to realize in the clinical stage in China owing to the lower medical level and the undeveloped economy. This assessment tool has been extensively adopted in China, but reliability and validity tests with large sample sizes are still needed.

It is still a challenge to estimate the risk thresholds, prevent and treat pregnancy-associated VTE [28, 29]. China still lacks systematic, effective VTE risk assessment tools, especially for pregnancy [30]. If a general VTE assessment scale is used for pregnant women, the risk of VTE to pregnant women is often underestimated, resulting in serious consequences. There have existed some advanced perinatal VTE assessment scales in western countries but they probably do not suit for Chinese population owing to racial differences, genetic background, food culture and life traditional differences. The incidence of hereditary thrombophilia is associated with race [31–37]. The lifestyle that the Chinese pregnant women spend the first month after giving birth is definitely different from that of women in western countries. Pregnant women live a confinement life during the first month and they will stay at home and will not do outdoor activities. Even in some rural areas, the pregnant women will lie in bed for one month and they seldom take showers. This kind of life style increases the risk to develop VTE. Therefore, the VTE tools used in western countries probably may not be applicable to the Chinese population. We have developed a risk assessment scale for perinatal venous thromboembolism in Chinese women using a Delphi‑AHP approach [38]. The Delphi method is a structured communication technique that uses successive rounds of questionnaires and evaluation by a panel of experts to reach a consensus on proposed items. The Analytic Hierarchy Process (AHP), developed by Saaty in the late 1970s, is one of the methods for multi-criteria decision-making. A Delphi-AHP approach uses both Delphi and AHP, which combines the advantages of Delphi and AHP [39]. However, our last scale still has some limitations. This study is a single-centered study only in Qingdao where the results probably cannot represent the whole Chinese population. The main reasons are as follows. First, the tool is a single-centered study only in Qingdao and Qingdao is a coastal city with a high economic development level and high education level located in the east of China. However, because of the huge area of China, the economic and education levels are imbalanced, and most cities in East China are developed and West China undeveloped. So the pregnant women in Qingdao probably cannot represent the whole situation of the whole country. Second, there are 56 nationalities in the whole of China and people with different nationalities have different lifestyles, which probably can affect the incidence of VTE. So, it is imperative to carry out a multi-center research with enough big sample size. The item pools of the scale were developed according to literature retrieval. The next one is a particular limitation of the Delphi expert method itself. The experts’ opinions included may probably be different from those of experts who were excluded during the questionnaire survey. Given the above two limitations, it is imperative to carry out a multi-center research with enough big sample size.

In this study, a retrospective study was conducted among pregnant women who delivered and developed a VTE in 12 hospitals all over China with a total of 1152 participants enrolled. A logistic regression method was conducted to determine the risk factors of VTE in women after full-term delivery. A fast assessment scale for perinatal VTE was developed, which possessed advantages of excellent sensitivity, specificity, Yuden index, and area under the ROC curve. The risk factor assessment scale for VTE in full-term pregnant women could distinguish the low-risk group from the high-risk group with good discrimination.

## Methods

### Participants

A retrospective study was conducted among pregnant women who delivered and caught complications of VTE in 12 hospitals, which are comparable in terms of level and size, all over China between January 2019 and January 2022. The hospitals were selected from east China, west China, south China, north China, and middle China, which represent the developed and undeveloped areas. The pregnant women were divided into an experimental group (VTE group) and a control group (without VTE). The ratio of pregnant women number in VTE to that in the control group was 1: 2. The sample size in each hospital is 96, including 32 cases with VTE and 64 cases without VTE. The samples were taken at random if they fit within a certain period (between January 2019 and January 2022). The incidence of VTE during pregnancy is approximately 1–2 per 1000 deliveries, which is consistent with that of all over China in the literature. There is no significant difference in the included 12 hospitals. The women in the control group were selected among mothers without VTE who gave birth adjacent delivery time of 1 week. The number of VTE and control group was 384 and 768, respectively. Inclusion criteria: Pregnant women were included in the study who experienced deep vein color Doppler ultrasonography check for both lower limbs before and after delivery and did not experience anticoagulation treatment during the perinatal period. Exclusion criteria: ① Pregnant women diagnosed with VTE before delivery; ②Twin and multiplets mother ③ Foreign parturients in hospital. Diagnosis standard of DVT patients: DVT of the lower extremity was confirmed by color Doppler ultrasonography. Pulmonary embolism diagnostic criteria: Pulmonary embolism was diagnosed by pulmonary CT examination.

### Design

The research group used a nurse’s workstation information system and maternal health records to retrospectively collect the participants’ personal information, including age, BMI before pregnancy, BMI before delivery, number of pregnancies, number of births, mode of conception, delivery mode, newborn weight, postpartum bleeding, gestational diabetes, gestational hypertension, thrombosis history, family history (thrombosis, diabetes, cardiovascular diseases), D-dimer values before delivery. According to the guidelines for weight gain issued by the USA Institute of Medicine in 2009, the maternal weight gain rate was divided into slow, normal, or fast [40].

The classification criterion: The criterion of differentiation is: BMI before pregnancy < 18.5 (low weight), BMI before pregnancy 18.5–24.9 (normal weight), BMI before pregnancy 25.0-29.9 (overweight), BMI before pregnancy ≥ 30.0 (obesity). For the pregnant women mentioned above, the upper and lower limits of weight gain rate in middle and late pregnancy were 0.58 − 0.44 kg/ week, 0.50-0.35 kg/ week, 0.33 − 0.23 kg/ week, 0.27 − 0.17 kg/week, respectively. Those whose weight gain rate is smaller than the lower limit are assigned to the slow group, those whose weight gain rate is higher than the upper limit are classified into the fast group, and the rest are assigned to the normal group [41]. All the above risk factors were analyzed using univariate analysis and multivariate analysis via a logistic regression model.

Finally, a facile rapid assessment scale for perinatal VTE in puerperium was developed based on the results of a multi-factor analysis. The scale was formed basing the Caprini thrombosis evaluation form updated in 2019 [42], thrombosis guidelines issued by the American College of Obstetricians and Gynecologists [36] and a document published by the Royal Society of Obstetricians and Gynaecologists [3, 43]. All the pregnant women who participated in the research were scored using the scale to testify their validity and effectiveness.

### Variables and definitions

The main variables include maternal age, BMI, mode of delivery, mode of fertilization, maternal thrombosis history, family history (thrombosis, diabetes, cardiovascular), weight gain rate during pregnancy, postpartum blood loss, gestational hypertension, gestational diabetes, number of pregnancy, number of delivery, D-dimer before delivery, and weight of the newborn. The body mass index (BMI) is used for defining height/weight characteristics in adults, which represents an index of an individual’s fatness [44]. Mode of delivery includes vaginal delivery, planned cesarean section and emergency cesarean section. Mode of fertilization includes natural conceptionand artificial insemination. The formula for weight gain during pregnancy is as below. Maternal weight gain rate (kg/week) = total weight gain during pregnancy (kg)/ gestational week (week) [40].

### Data analysis

SPSS 19.0 statistical software was used for data process and statistics analysis. The counting data were expressed by percentage, and tested through single-factor analysis using chi-square and standard deviation. To avoid missing important risk factors, the single factor with P < 0.2 was taken into the regression equation. Multivariate analysis was performed using a logistic regression model and P < 0.05 was considered as a statistically significant difference. A T-test of two independent samples was used to evaluate the scale discrimination. The prediction effect of the scale was judged by sensitivity, specificity, Yuden index and area under the ROC curve. The test level of the prediction effect of the scale was α = 0.05 [39, 45]. The Hosmer-Lemeshow test, concordance index (C-index), and calibration curve were also used to evaluate the scale.

### Ethical consideration

The study was approved by the Ethics Committee of the Affiliated Hospital of Qingdao University, China. The institutional review board has approved the study and waived the need for individual informed consent by formulating a declaration of no objection.

## Results

### Pregnant women’s demographic characteristics

A total of 1152 parturients were enrolled in this study, including 826 primiparas (71.7%) and 326 parturients (28.3%). The age ranges from 23 to 45 (30.25 ± 3.65) years old. Pregnancy days are 245 ~ 290 (274.85 ± 9.58) days. Education: 362 colleges or below (31.4%), 490 bachelors (42.5%), 300 masters or above (26.1%). In this study, the ratio of pregnant women with VTE to those without VTE is 1/2, with 384 women (33.3%) and 768 women without VTE (66.7%). The sample size is 1152, which is sufficient for the statistical study [46]. The power of a statistical test of a null hypothesis (H0) is the probability that the H0 will be rejected when it is false, that is, the probability of obtaining statistically significant results. Statistical power (1-β) depends on the significance criterion (ɑ), the sample size(N), and the population effect size (ES) [47]. In this study, the statistical power is 0.95 which is calculated by G*power software (ɑ=0.05).

### Univariate analysis

The two groups have a statistically significant difference in age, BMI before delivery, delivery mode, family history (thrombosis, diabetes, cardiovascular disease), and the number of pregnancies (P < 0.05), while there are no significant differences in pre-pregnancy BMI, gestational weight gain rate, parturition, postpartum blood loss, gestational hypertension, gestational diabetes, previous history of thrombosis, mode of conception, plasma D-dimer value before delivery, and newborn birth weight (P > 0.05), as shown in Table 1.

**Table 1:** Single factors of scale for VTE [ n (%)]

Characteristics		VTE group (n = 384)	Control group (n = 768)	χ^2^	P
Age (years)	< 35	175(45.6)	602(78.4)	8.685	0.003
	≥ 35	208(54.2)	166(21.6)		
BMI before pregnant	<18.5	20(5.2)	108(14.1)	3.658	0.300
	18.5~	327(85.2)	538(70.1)		
	25.0~	20(5.2)	111(14.4)		
	≥ 30.0	12(3.1)	11(1.4)		
BMI before delivery	18.5~	43(11.2)	273(35.6)	7.768	0,020
	25.0~	250(65.1)	286(37.2)		
	≥ 30.0	91(23.7)	209(27.2)		
Weight gain rate during pregnancy	slow	33(8.5)	67(8.7)	1.396	0.482
	normal	132(34.4)	350(45.6)		
	fast	219(57.1)	351(45.7)		
Delivery way	vaginal delivery	0	230 (30.0)	11.356	0.008
	planned cesarean section	220 (57.2)	363 (47.2)		
	emergency cesarean section	164 (42.8)	175 (22.8)		
Postpartum blood loss (ml)	< 500 ml for vaginal delivery or < 1 000 ml for cesarean delivery	384 (100)	723(94.2)		0.550(F)
	≥ 500 ml for vaginal delivery or ≥ 1 000 ml for cesarean	0	45 (5.8)		
Gestational hypertension	no	351 (91.5)	647(84.2)	2.238	0.324
	yes	33 (8.5)	121 (15.8)		
Gestational diabetes	no	341 (88.7)	582 (75.8)	1.968	0.162
	yes	43 (11.3)	186 (24.2)		
Family history (thrombosis, diabetes, cardiovascular)	no	186 (48.5)	603 (78.5)	7.584	0.005
	yes	198 (51.5)	165 (21.5)		
Maternal thrombosis history	no	362 (94.2)	768 (100)	-	0.355 (F)
	yes	22 (5.8)	0 (0)		
Number of pregnancy (n)	<3	241 (62.8)	681 (88.7)	7.690	0.005
	≥ 3	143 (37.2)	87 (11.3)		
Number of delivery (n)	1	275 (71.5)	624 (81.3)	2.003	0.359
	2	109 (28.5)	121 (15.7)		
	≥ 3	0 (0)	23 (3.0)		
Fertilization way	natural conception	362 (94.4)	636 (82.8)	1.358	0.196
	artificial insemination	22 (5.6)	132 (17.2)		
D-dimer before delivery (mg/L)	≤ 3.05	253 (65.8)	626 (81.5)	1.962	0.160
	> 3.05	131 (34.2)	142(18.5)		
Weight of newborn (g)	<2 500	22 (5.8)	43 (5.6)	0.360	0.780
	2 500~	308 (80.1)	657 (85.6)		
	>4 000	54 (14.1)	68 (8.8)		

### Multivariate analysis

A multi-factor analysis was conducted, taking-term pregnant women suffering VTE or not as the dependent variable and the variable with P < 0.2 in the univariate analysis as the independent variable. Generally, variables with P < 0.05 or p < 0.2 can be included in the multivariate analysis. The advantage of choosing p < 0.2 can avoid missing some important variables. The assignment point for each factor is as follows. Age: 0 points for age<35, 1 point for age ≥ 35; BMI before delivery: 0 points for 18. 5 ~ 24.9, 1 point for 25.0 ~ 29.9, 3 points for ≥ 30.0. Delivery way: 0 points = vaginal delivery, 1 point = planned cesarean section, 2 points = emergency cesarean section. Gestational diabetes: 0 point = no, 1 point = yes;Family history (thrombosis, diabetes, cardiovascular):0 = no, 1 = yes; Number of pregnancy: 0=<3, 1 = ≥ 3; Fertilization way: 0 = natural conception, 1 = artificial insemination. D-dimer before delivery (mg/L): 0 = ≤ 3.05, 1=>3.05. Logistic analysis shows that age ≥ 35 years, BMI ≥ 30.0 before delivery, gestational diabetes, family history (thrombosis, diabetes, cardiovascular diseases) and artificial insemination are risk factors for VTE in full-term pregnant women (P < 0.05). The Logistic regression analysis results are shown in Table 2.

**Table 2:** Logistic regression analysis of risk factors for VTE

variable		Regression coefficient	Standard error	Wald χ2	P	OR	95% CI
constant		-0.686	0.232	9.30	<0.05	0.190	0.015 ~ 0.592
Age (years)	≥ 35.0	-2.502	0.983	6.250	0.014	0.088	0.036 ~ 2.801
BMI before delivery	25.0 ~ 30.0	-1.096	1.122	1.108	0.289	0.312	1.700 ~ 111.235
	≥ 30.0	2.582	1.101	6.040	0.015	13.852	0.003 ~ 2.205
Delivery way	planed cesarean	-2.045	2.112	0.001	1.000	0.128	334.008 ~ 4
	emergency cesarean	9.902	1.862	0.001	0.099	474.020	754.006
gestational diabetes	yes	3.802	1.601	5.680	0.018	44.480	1.994 ~ 990.4355
Family history (diabetes, cardiovascular disease)	yes	-1.798	0.903	4.225	0.038	0.165	0.029 ~ 0.935
Delivery times	≥ 3	-1.504	1.023	2.114	0.150	0.226	0.014 ~ 1.802
Fertilization mode	assisted productive	2.259	1.108	4.405	0.040	9.698	1.142 ~ 84.465
D- dimer before delivery (mg/L)	> 3.05	-1.102	0.845	1.538	0.225	0.366	0.072 ~ 1.862

### Development of a fast assessment scale for perinatal VTE

According to the Logistic regression analysis results, age, BMI before delivery, gestational diabetes, family history and conception mode were included in the scoring scale. Due to the small sample size included in this study, some important factors probably were removed during the univariate analysis. However, the previous history of thrombosis is the most important factor affecting the occurrence of VTE after childbirth and the risk of VTE after cesarean section, especially for emergency cesarean section, is also significantly increased, which has been mentioned in Thromboembolism in Pregnancy Practice Bulletin issued by American College of Obstetricians and Gynecologists [36] and Royal College of Obstetricians and Gynaecologists (RCOG)[43]. Special attention should be paid to the risk of VTE in pregnant women with hypertension, especially in women with preeclampsia. Therefore, based on the research results and the guidance, the items in the VTE scale for rapid evaluation of postpartum VTE in full-term pregnant women were determined. A total of 1152 parturients were enrolled in this study, including 826 primiparas (71.7%) and 326 parturients (28.3%). So the sample size is enough for statistical analysis. So this study was not underpowered [46].

The scoring standard of the brief scale is established according to the type of variables. (1) if risk factors are dichotomous variables: The value is assigned 1 point when the risk factor exists, and assigned 0 points when it does not exist. (2) if the risk factors were ordered multivariate variables: The value is assigned 0 points when the variable is graded to level 1, 1 point for level 2, and 2 points for level 4. Maternal thrombosis history and family history of thrombosis are widely accepted as the most important factors for VTE, the assignment value of the maternal or family history of VTE is adjusted to 2 points (See Table 3).

**Table 3:** Risk factors assessment scale for VTE

Characteristics	0 point	1 point	2 points
Age (years)	< 35	≥ 35	-
BMI before delivery	<25	25 ~ 29.9	≥ 30
Fertilization mode	natural conception	assisted reproductive	-
Delivery mode	vaginal delivery	planned cesarean section	emergency cesarean section
Gestational diabetes	no	yes	-
Gestational hypertension	no	yes	-
Maternal VTE history	no	-	yes
Family history	no	diabetes, cardiovascular disease	VTE

### Discrimination of the scale

All the 1152 pregnant women included in the study were scored according to the VTE scale and ranked in ascending order. The last 25% and the top 25% were named as a low-risk group and a high-risk group, respectively. Based on the scoring sequence, the low and high-risk boundary points were determined, with a low risk of 1 point and a highrisk of 4 points. Combing the generally used Caprini scale, a score of below 1 point was classified as low risk, 2 as medium risk, and 3 ~ 4 as high risk, ≥ 5 as extremely high risk. The differences between the high-risk group and the low-risk group were compared to determine whether the scale could effectively distinguish patients in the low-risk group and high-risk group when taking the standard values of all sectors. The results showed that 285 patients in the low-risk group scored 0.56 ± 0.51 points and 384 cases in the high-risk group with a score of 5.05 ± 1.12 points. The difference between the two groups was statistically significant (P < 0.001), indicating that the risk factor assessment scale for VTE in full-term pregnant women could distinguish the low-risk group from the high-risk group with good discrimination. The scale is of high prediction efficiency and effectiveness in the rapid evaluation of postpartum VTE in full-term pregnant women. The scale has high discrimination, indicating that the risk factor assessment scale for VTE could distinguish the low-risk group from the high-risk group.

### Efficiency and effectiveness of the scale

**Fig. 1:**
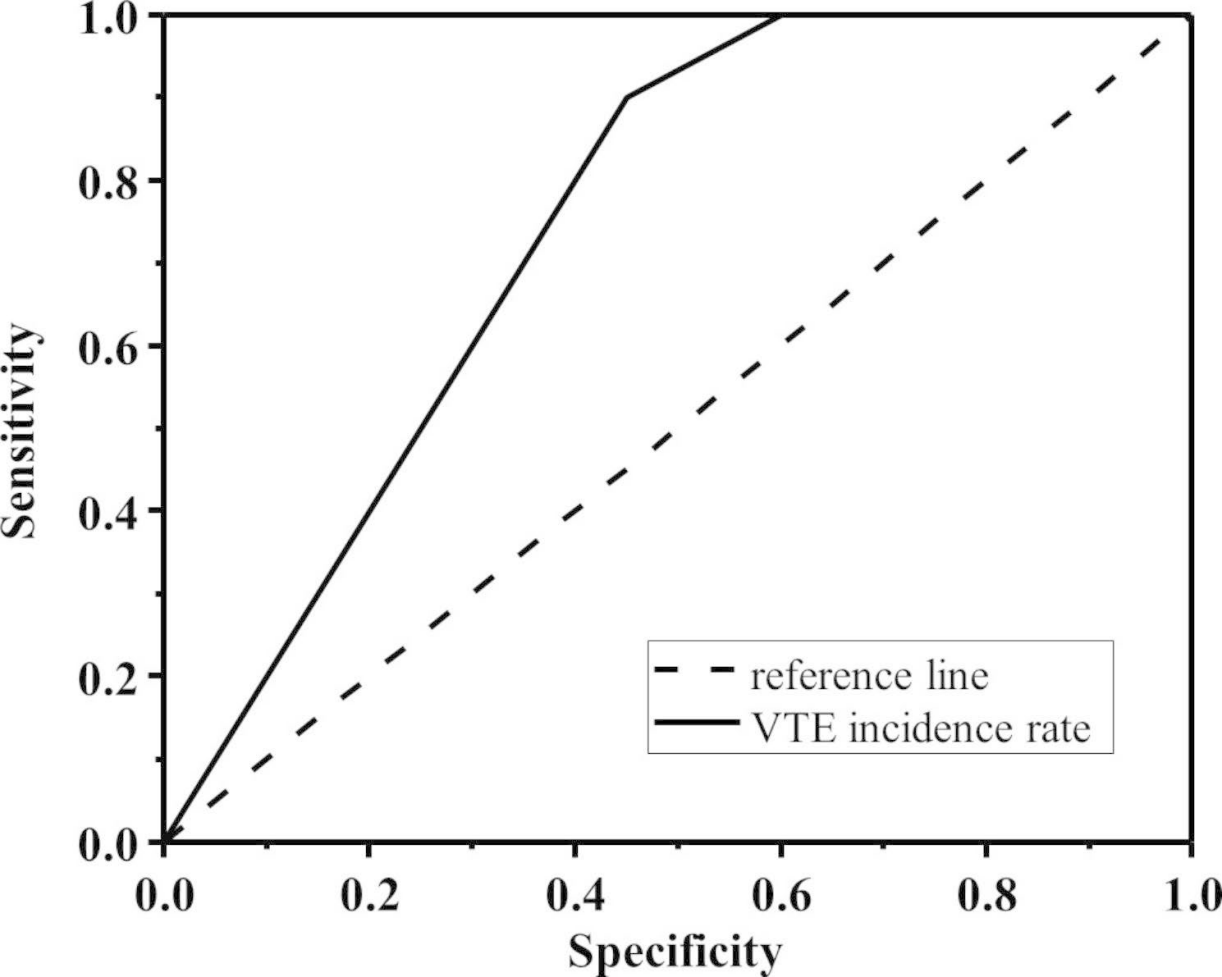
ROC curve of scale assessment

**Fig. 2:**
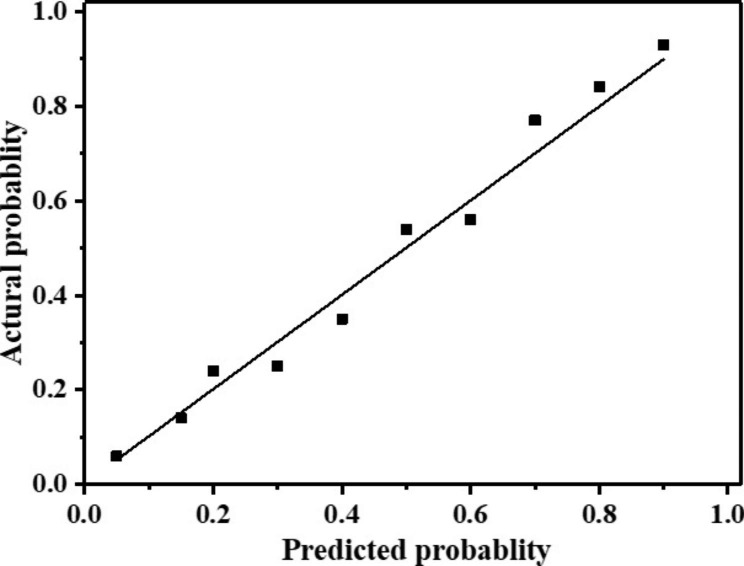
Calibration curve

In this study, the ROC curve was used to test the prediction efficiency and effectiveness of the short scale for rapid evaluation of postpartum VTE in full-term pregnant women. The test indexes included sensitivity, specificity, Yuden index, and area under the ROC curve (AUC). The results showed that the sensitivity, specificity, Yuden index, and area under the ROC curve were 0.578, 0.756, 0.334 and 0.878, respectively, as shown in Fig. 1. The Hosmer-Lemeshow test, concordance index (C-index), and calibration curve were also used to evaluate the goodness and prediction accuracy of the scale. The Hosmer-Lemeshow test (HL test) is a model fitting index, which is used to judge the gap between the predicted value and the real value. In this study, p > 0.05 indicates that the HL test is passed and there is no significant difference between the predicted value and the true value. The C-index value is 0.88, which means that the probability that the predicted result agrees with the actual result is 88%. The calibration curve is a visualization of the results of the goodness of Fit test of the Hosmer-Lemeshow, which is often used to evaluate the logistic regression model. The calibration curve showed that the scale’s actual probability is very close to the predicted probability (Fig. 2).

## Discussion

The risk of postpartum venous thromboembolism in elderly women was higher. It was reported that the incidence of VTE for pregnant women older than 35 in puerpera was higher the younger women. The incidence rate increases 2–10 times with the increase in age. In this research, the proportion of elderly mothers in the VTE group was 52.5%. This may be due to the reasons that the elderly women missed the best reproductive opportunity, they were easy to cause a variety of complications and amalgamation and blood viscosity was higher in 3 h after delivery. Therefore, nursing staff should pay attention to the prediction of high-risk VTE in elderly women (with age ≥ 35 years) and take care of maternal complaints and timely detect discomfort and anticipate the risk of VTE.

Obese pregnant women are easily complicated with venous thromboembolism after delivery. Abnormal coagulation system and platelet function excessive active in obese patients are easy to induce a hypercoagulable condition. Research showed that the possibility of VTE incidence increased 2-5-fold if the pregnant BMI ≥ 25. The risk for VTE in pregnant women with BMI ≥ 30 was 14.0 times higher than in mothers with BMI < 25. During pregnancy education and perinatal medical examination, the medical staff should underline the importance of a healthy diet and moderate exercise, which can help pregnant women to control their weight reasonably during pregnancy and reduce the risk of postpartum VTE [48, 49].

Pregnant women with gestational diabetes mellitus or gestational hypertension are the population with a high risk of venous thromboembolism. The results of this study showed that the incidence of VTE in pregnant women with gestational diabetes was 46.0 times that in normal women without VTE. The latest Caprini thrombosis assessment scale also lists diabetes as an important influencing factor for VTE [42]. In addition, gestational hypertension, especially preeclampsia and eclampsia, is widely recognized as one of the high-risk factors for postpartum VTE in the world. The coagulation factor of pregnant women with hypertension significantly increased and the fibrinolytic activity was relatively weak, which lead to coagulation system disorder and increase the risk of postpartum VTE [50]. So it is necessary to strengthen the prediction of VTE risk in pregnant women with gestational diabetes mellitus and gestational hypertension to reduce the risk of VTE incidence.

Assisted reproductive technology is the potential risk factor for venous thromboembolism in postpartum [51]. According to statistics, the incidence of VTE after ovulation induction using exogenous gonadotropin was about 0.04%. This study also shows that the VTE risk among pregnant women with artificial insemination is 10.0 times higher than that of pregnant women with natural conception. This probably is related to the complications which are induced by assisted reproductive technology. Ovarian hyperstimulation syndrome is the main complication of assisted reproductive technology, which leads to hypercoagulability, slow blood flow, and finally the incidence of VTE. At present, obstetricians and nurses do not pay enough attention to the occurrence of VTE in pregnant women with assisted reproductive technology in China. Therefore, early diagnosing, early prevention, and early warning should be conducted in clinical work.

It was reported that genetic factor is an important factor for deep venous thrombosis (DVT) [52]. When considering the influence of family history on VTE, diabetes and cardiovascular diseases were included as risk factors in the VTE assessment scale. Studies show that a history of thrombosis is the most dangerous factor for the occurrence of VTE and the risk of recurrence of VTE increases 3 ~ 4 times [53–56]. In this study, only 1 woman had a history of thrombosis, and this influencing factor is not included in the final scale, which is probably due to the insufficient sample size of this study. Family history is an important component of maternal history for risk assessment of VTE. During the risk assessment of VTE, the inquiry of maternal family history should be strengthened. To avoid the omission of relevant content in the medical history record, it is recommended that the nurse take face-to-face inquiry during the evaluation of family history to ensure that all relevant family history is included in the risk assessment.

In the scale, the index used to predict the risk of VTE includes sensitivity, specificity, the Yuden index, the area under the ROC curve, etc. According to the results, if the scale is classified based on 1 point and 4 points, the sensitivity is 0.578 and the specificity is 0.756. If the area under the ROC curve of 0.878, and the prediction efficiency is medium. Overall, the rapid VTE assessment scale in postpartum is useful to predict the incidence risk for VTE.

There are some strengths and limitations to this study. The first strength is that a sufficiently big sample size of participants was conducted and the participants were from 12 cities in developed and undeveloped areas, which guaranteed representativeness. The second strength is that a retrospective case-control study was used in this research and a logistic regression method was conducted to determine the risk factors of VTE. It guarantees the assessment scale for perinatal VTE of scientificity, validity, and reliability.

## Conclusions

In conclusion, maternal age, BMI before delivery, gestational diabetes mellitus, mode of conception, and family history of related diseases were the main independent risk factors for VTE in puerperium in Chinese pregnant women. Based on our research results, a fast assessment scale for perinatal VTE was developed, which possessed advantages of excellent sensitivity, specificity, Yuden index, and area under the ROC curve.

## Data Availability

The authors confirm that the data supporting the findings of this study are available within the article.

## References

[1] Kaaja, Risto J, Galambosi, Paivi J, Gissler, Mika, Ulander V-M (2017). Incidence and risk factors of venous thromboembolism during postpartum period: a population-based cohort-study. Acta Obstet et Gynecol Scandinavica: Official Publication Nordisk Forening Obstetrik och Gynekologi.

[2] Lewis G, editor. Saving Mothers’ Lives. The Eighth Report of Confidential Enquiries into Maternal Deaths in the United Kingdom. London. 2011.

[3] RCOG: Thromboembolic Disease in Pregnancy and the Puerperium: Acute Management. Green-top Guideline No. 37b. 2015:https://www.rcog.org.uk/globalassets/documents/guidelines/gtg-37b.pdf.

[4] Blondon M, Tejada B, Glauser F, Righini M, Robert-Ebadi H (2021). Management of high-risk pulmonary embolism in pregnancy. Thromb Res.

[5] Evensen LH, Isaksen T, Brækkan SK, Hansen JB. Physical activity and risk of recurrence and mortality after incident venous thromboembolism. J Thromb Haemost 2019. 10.1111/jth.14449 30985982

[6] Virkus RA, L?Kkegaard ECL, Bergholt T, Mogensen U, Langhoff-Roos J, Lidegaard. Venous thromboembolism in pregnant and puerperal women in Denmark 1995–2005. A national cohort study. Thromb Haemostasis. 2011;105(2):304–9. 10.1160/TH10-12-0823 21713323

[7] Sefogah PE, Nuamah MA, Swarray-Deen A, Mumuni K, Onuzo CN, Seffah JD (2021). Venous thromboembolism risk and prophylaxis in hospitalized obstetric patients at a tertiary hospital in Accra, Ghana: a comparative cross-sectional study. Int J Gynecol Obstet.

[8] Calderwood CJ, Thanoon OI (2013). Venous thromboembolism in pregnancy. Obstet Gynecol Reproductive Med.

[9] Chan LY, Tam WH, Lau TK (2001). Venous thromboembolism in pregnant chinese women. Obstet Gynecol.

[10] Folkeringa N, Brouwer J, Korteweg FJ, Veeger N, Erwich J, Meer J (2007). High risk of pregnancy-related venous thromboembolism in women with multiple thrombophilic defects. Br J Haematol.

[11] Roopen SVLNPJPA (2018). Venous thromboembolism and women’s health. Br J Haematol.

[12] Ernst DM, Oporto JI, Zuniga PA, Pereira JI, Vera CM, Carvajal JA (2021). Maternal and perinatal outcomes of a venous thromboembolism high-risk cohort using a multidisciplinary treatment approach. Int J Gynecol Obstet.

[13] Andrew L, Inle FN, Blondon M, Rodger MA, Skeith L (2020). Preventing postpartum venous thromboembolism: a call to action to reduce undue maternal morbidity and mortality. Thromb Res.

[14] Morikawa M, Adachi T, Itakura A, Nii M, Nakabayashi Y, Kobayashi T (2021). A retrospective cohort study using a national surveillance questionnaire to investigate the characteristics of maternal venous thromboembolism in Japan in 2018. BMC Pregnancy Childbirth.

[15] Guimicheva B, Czuprynska J, Arya R (2015). The prevention of pregnancy-related venous thromboembolism. Br J Haematol.

[16] Chandra D, Dabhi K, Lester W. Are we assessing venous thromboembolism (VTE) risk appropriately for hospitalised medical patients? The National VTE Risk Assessment Tool versus Padua Prediction score. Br J Haematol 2020, 189(1). 10.1111/bjh.16411 31978942

[17] Amy S, Rachel R (2019). Venous thromboembolic risk assessment in pregnancy: comparison of the All-Wales maternity risk assessment tool with guidance from the Royal College of Obstetrics and Gynaecology. Br J Haematol.

[18] King A, D’Souza RD, Herman D, Malinowski AK (2020). Outcome reporting in studies on perinatal venous thromboembolism: a systematic review. Obstet Gynecol.

[19] Dargaud Y, Rugeri L, Ninet J, Negrier C, Trzeciak MC (2005). Management of pregnant women with increased risk of venous thrombosis. Int J Gynecol Obstet.

[20] Dargaud Y, Rugeri L, Vergnes MC, Arnuti B, Trzeciak MC (2010). A risk score for the management of pregnant women with increased risk of venous thromboembolism: a multicentre prospective study. Br J Haematol.

[21] Dargaud Y, Rugeri L, Fleury C, Battie C, Gaucherand P, Huissoud C, Rudigoz RC, Desmurs-Clavel H, Ninet J, Trzeciak MC. Personalized thromboprophylaxis using a risk score for the management of pregnancies with high risk of thrombosis: a prospective clinical study. J Thromb Haemostasis 2017. 10.1111/jth.13660 28231636

[22] Chauleur C, Quenet S, Varlet MN, Seffert P, Laporte S, Decousus H, Mismetti P (2008). Feasibility of an easy-to-use risk score in the prevention of venous thromboembolism and placental vascular complications in pregnant women: a prospective cohort of 2736 women. Thromb Res.

[23] Céline C, Jean-Christophe G, Silvy L, Céline C, Laurent B, Véronique E, Pascal G, Eva B, Olivier D, De Nis G. Benefit of risk score-guided prophylaxis in pregnant women at risk of thrombotic events: a controlled before-and-after implementation study. Thrombosis & Haemostasis; 2018. pp. –0038. 10.1055/s-0038-1668524 30103244

[24] Schoenbeck D, Nicolle A, Newbegin K, Hanley J, Loughney AD (2011). The Use of a Scoring System to Guide Thromboprophylaxis in a high-risk pregnant Population. Thrombosis.

[25] Sophie T, Serena PM, Oriana. Paoletti, Paolo, Bucciarelli, Enrica, Ronca: The “Pregnancy Health-care Program” for the prevention of venous thromboembolism in pregnancy. Internal&Emergency Med 2015. 10.1007/s11739-014-1111-6 25078669

[26] Shannon B. Ian, Greer, Saskia, Middeldorp, David: VTE, thrombophilia, antithrombotic therapy, and pregnancy: Antithrombotic Therapy and Prevention of Thrombosis, 9th ed: American College of Chest Physicians Evidence-Based Clinical Practice Guidelines. *Chest* 2012. 10.1378/chest.11-2300 PMC3278054 22315276

[27] Bates SM, Rajasekhar A, Middeldorp S, Mclintock C, Rodger MA, James AH, Vazquez SR, Greer IA, Riva JJ, Bhatt M. American Society of Hematology 2018 guidelines for management of venous thromboembolism: venous thromboembolism in the context of pregnancy *blood advances* 2018, 2(22):3317–59. 10.1182/bloodadvances.2018024802 PMC6258928 30482767

[28] Gassmann N, Viviano M, Righini M, Fontana P, Martinez de Tejada B, Blondon M (2021). Estimating the risk thresholds used by guidelines to recommend postpartum thromboprophylaxis. J Thromb Haemost.

[29] Federspiel JJ, Wein LE, Addae-Konadu KL, Darwin KC, Talamo LE, Myers ER, James AH (2021). Venous thromboembolism incidence among patients recommended for pharmacologic thromboembolism prophylaxis after cesarean delivery in selected guidelines. J Thromb haemostasis: JTH.

[30] Cai H, Liu J, Zhu Y, Feng S (2021). Research progress on risk assessment of pregnancy associated venous thromboembolism. Chin J Mod Nurs.

[31] Chan W-S. Diagnosis of venous thromboembolism in pregnancy. Thromb Res 2017:S0049384817304826. 10.1016/j.thromres.2017.09.003 28935434

[32] Chen Y, Dai Y, Song J, Wei L, Ma Y, Tian N, Wang Q, Zhang Q, Zhang Y, Wang XL. Establishment of a risk assessment tool for pregnancy-associated venous thromboembolism and its clinical application: protocol for a prospective observational study in Beijing. BMC Pregnancy Childbirth 2019, 19. 10.1186/s12884-019-2448-7 PMC6693270 31409379

[33] Cohen AT, Granziera S (2015). Excellence, quality and limitations of the NICE venous thromboembolism score tool: how can it be improved?. Br J Haematol.

[34] Butterworth K, Iyen B, Grainge MJ (2019). Venous thromboembolism and race: a systematic review and meta-analysis. Br J Haematol.

[35] Zhao Z, Zhou Q, Li X. Missed opportunities for venous thromboembolism prophylaxis during pregnancy and the postpartum period: evidence from mainland China in 2019. BMC Pregnancy Childbirth 2021, 21(1). 10.1186/s12884-021-03863-w PMC8142288 34030656

[36] ACOG (2018). ACOG Practice Bulletin No. 196 Summary: thromboembolism in pregnancy. Obstet Gynecol.

[37] Blondon M, Harrington LB, Righini M, Boehlen F (2014). Racial and ethnic differences in the risk of postpartum venous thromboembolism: a population-based, case-control study. J Thromb Haemost.

[38] Zhang M, Liu M, Wang D, Wang Y, Zhang W, Yang H, Zhang J, Li Q, Guo Z (2022). Development of a risk assessment scale for perinatal venous thromboembolism in chinese women using a Delphi-AHP approach. BMC Pregnancy Childbirth.

[39] Zhang M, Chen W, Liu C, Sui J, Wang D, Wang Y, Meng X, Wang Y, Yue C (2021). Nursing-sensitive quality indicators for pernicious placenta previa in obstetrics: a Delphi study based across chinese institutions. Nurs Open.

[40] Rasmussen K, Yaktine A (2009). Institute of Medicine (US). Committee to reexamine IOM pregnancy weight guidelines. Weight gain during pregnancy: reexamining the Guidelines.

[41] Li M (2018). Relationship among Prepregnancy BMI, gestational weight gain and gestational blood pressure.

[42] Cronin MA, Dengler N, Krauss ES, Segal A, Caprini JA (2019). Completion of the updated Caprini Risk Assessment Model (2013 version). Clin Appl Thromb Hemost.

[43] RCOG. : Reducing the risk of thrombosis and embolism during pregnancy and the puerperium. 2019.

[44] Universityofminnesota M. Body mass index: obesity, BMI, and health: a critical review. Nutr Today 2015(50 – 3). 10.1097/NT.0000000000000092 PMC4890841 27340299

[45] Zhang M, Chen W, Liu C, Wang D, Liu L, Yuan X, ChongyuYue (2021). Construction and analysis of nursing quality sensitive indicators for vaginal birth after cesarean in obstetrics. J Nurs (China).

[46] Burmeister E, Aitken LM (2012). Sample size: how many is enough?. Australian Crit Care.

[47] Cohen J (1992). Statistical power analysis. J Roy Stat Soc.

[48] Malinowski AK, Bomba-Opoń D, Parrish J, Sarzyńska U, Dan F (2017). Venous thromboembolism in obese pregnant women: Approach to diagnosis and management. Ginekologia polska.

[49] Ads AE, Acg A, Ahj A, Hka B (2020). Developing a model for predicting venous thromboembolism in obese pregnant women in a national study - ScienceDirect. Thromb Res.

[50] Scheres LJJ, Lijfering WM, Groenewegen NFM, Koole S, de Groot CJM, Middeldorp S, Cannegieter SC (2020). Hypertensive complications of pregnancy and risk of venous thromboembolism. Hypertension.

[51] Goualou M, Noumegni S, Moreuil CD, Guillou ML, Coninck GD, Clément.Hoffmann, Robin S, Morcel K, Moigne EL, Tremouilhac C (2022). Venous Thromboembolism Associated with assisted Reproductive Technology: a systematic review and Meta-analysis. Thromb Haemost.

[52] Li Q, Kai Y (2010). Improved correction for population stratification in genome-wide association studies by identifying hidden population structures. Genet Epidemiol.

[53] Lazo-Langner A, Al-Ani F, Weisz S, Rozanski C, Louzada M, Kovacs J, Kovacs MJ. Prevention of venous thromboembolism in pregnant patients with a history of venous thromboembolic disease: a retrospective cohort study. Thromb Res 2018:20–5. 10.1016/j.thromres.2018.05.005 29772489

[54] Nagler M, Kuijk S, Cate HT, Prins MH, Cate-Hoek A. Predicting recurrent venous thromboembolism in patients with deep-vein thrombosis: Development and Internal Validation of a potential New Prediction Model (Continu-8). Front Cardiovasc Med, 8:655226. 10.3389/fcvm.2021.655226 PMC8055939 33889600

[55] Natae SF, Kosa Z, Sandor J, Merzah MA, Bereczky Z, Piko P, Adany R, Fiatal S. The higher prevalence of venous thromboembolism in the hungarian Roma Population could be due to elevated genetic risk and stronger gene-environmental interactions. Front Cardiovasc Med 2021, 8. 10.3389/fcvm.2021.647416 PMC8576195 34765649

[56] Hindberg K, Isaksen T, Braekkan S, Hansen K (2017). Recurrence and mortality after first venous thromboembolism in a large population-based cohort. J Thromb haemostasis: JTH.

